# The DSIF Subunits Spt4 and Spt5 Have Distinct Roles at Various Phases of Immunoglobulin Class Switch Recombination

**DOI:** 10.1371/journal.pgen.1002675

**Published:** 2012-04-26

**Authors:** Andre Stanlie, Nasim A. Begum, Hideo Akiyama, Tasuku Honjo

**Affiliations:** 1 Department of Immunology and Genomic Medicine, Graduate School of Medicine, Kyoto University, Kyoto, Japan; 2 New Frontiers Research Laboratories, Toray Industries, Kanagawa, Japan; The University of North Carolina at Chapel Hill, United States of America

## Abstract

Class-switch recombination (CSR), induced by activation-induced cytidine deaminase (AID), can be divided into two phases: DNA cleavage of the switch (S) regions and the joining of the cleaved ends of the different S regions. Here, we show that the DSIF complex (Spt4 and Spt5), a transcription elongation factor, is required for CSR in a switch-proficient B cell line CH12F3-2A cells, and Spt4 and Spt5 carry out independent functions in CSR. While neither Spt4 nor Spt5 is required for transcription of S regions and AID, expression array analysis suggests that Spt4 and Spt5 regulate a distinct subset of transcripts in CH12F3-2A cells. Curiously, Spt4 is critically important in suppressing cryptic transcription initiating from the intronic Sμ region. Depletion of Spt5 reduced the H3K4me3 level and DNA cleavage at the Sα region, whereas Spt4 knockdown did not perturb the H3K4me3 status and S region cleavage. H3K4me3 modification level thus correlated well with the DNA breakage efficiency. Therefore we conclude that Spt5 plays a role similar to the histone chaperone FACT complex that regulates H3K4me3 modification and DNA cleavage in CSR. Since Spt4 is not involved in the DNA cleavage step, we suspected that Spt4 might be required for DNA repair in CSR. We examined whether Spt4 or Spt5 is essential in non-homologous end joining (NHEJ) and homologous recombination (HR) as CSR utilizes general repair pathways. Both Spt4 and Spt5 are required for NHEJ and HR as determined by assay systems using synthetic repair substrates that are actively transcribed even in the absence of Spt4 and Spt5. Taken together, Spt4 and Spt5 can function independently in multiple transcription-coupled steps of CSR.

## Author Summary

Class switch recombination (CSR) in B cells is required for interaction with different effector molecules while retaining the affinity for the same antigens. CSR mechanism involves the orchestrated steps of transcription, DNA break, and repair of the target loci. Within the cells, these processes occur at the chromatin level—involving DNA, histones, and their associated post-translational modifications (PTMs). Transcription factors associated with RNA Polymerase II complex often have regulatory roles in chromatin maintenance, which in turn might regulate the process of DNA cleavage and repair. Here we report that the transcription factor DSIF complex (Spt4 and Spt5) is critically required for CSR. The absence of either Spt4 or Spt5 blocked CSR. Interestingly, Spt4 and Spt5, although previously thought to work as a complex, can function independently of each other at several nodes of CSR, namely transcription regulation, DNA break formation, and histone PTM maintenance, exemplified by H3K4me3. The importance of H3K4me3 unifies three programmed recombinations—CSR, VDJ, and meiotic—in their reliance on this modification for their respective DNA cleavage formations. Moreover, Spt4 and Spt5 are required for DNA repair, another critical aspect of CSR, suggesting that the DNA repair steps of CSR may be coupled with transcription.

## Introduction

Immunoglobulin (Ig) class switch recombination (CSR), which takes place in activated B lymphocytes, alters antibody effector functions by changing the Ig heavy-chain constant region (C_H_) from Cμ (IgM) to other C_H_s (namely, IgGs, IgE, or IgA). CSR is initiated by the cleavage of two DNA switch (S) regions, a donor and an acceptor locus, located 5′ to each C_H_ region [1]. S region double-strand breaks (DSBs), which are generated by staggered nicks, are paired and recombined by the general repair mechanisms; non-homologous end joining (NHEJ) or alternative end joining [2], [3], [4], [5]. Simultaneously, the intervening C_H_ region between the donor and acceptor S regions is looped out and deleted [1].

CSR absolutely depends on three critical events: (a) the expression of activation-induced cytidine deaminase (AID), a master regulator of Ig gene diversification processes including CSR, somatic hypermutation (SHM), and gene conversion [6], [7], [8], [9]; (b) the active transcription of S regions, which contain highly repetitive sequences [10], [11]; and (c) the repair and joining of the cleaved DNA ends [5]. The requirement of AID in CSR was convincingly shown by the finding that both *Aicda* knockout model mice and human patients with *AICDA* mutations fail to produce Ig isotypes other than IgM [6], [7]. Subsequent AID-mutant studies showed that AID controls two CSR intermediate steps; mutations in the AID N-terminal region affect cleavage of both the S and variable (V) regions, while mutations in the C-terminal domain are capable of cleaving DNA but incapable of recombining the cleaved S regions [12], [13], [14].

Active S region transcription has been associated with efficient CSR [15], [16], and its absolute requirement was demonstrated by gene-targeting experiments [10], [11]. S region transcription is initiated from the I promoter located upstream of each S region, and terminates downstream of the C_H_ region. The mature transcripts, designated as germline transcripts (GLTs), contain the I and C_H_ exons after splicing out the S region and the C_H_ intronic sequences [15], [16]. Active S region transcriptions are predicted to readily form non-B DNA structures due to the repetitive nature of the S region sequences [17],[18],[19],[20]. Indeed, decreasing the amount of topoisomerase 1 (Top1) protein appears to cause excessive negative supercoiling to accumulate behind the transcription machinery, which could facilitate the formation of non-B DNA structures within repetitive sequences in the S regions and triplet repeats; the latter is implicated in Huntington's disease [19], [21], [22]. These unusual DNA structures are proposed to be suitable substrates for Top1-mediated irreversible cleavage [19], [21]. According to the DNA deamination model, transcription-induced DNA structural alteration such as R-loop formation is proposed to be critical for AID to directly deaminate S region cytosine [23], [24], [25].

Transcription by RNA polymerase II (RNAPII) through chromatin is associated with various histone post-translational modifications (PTMs) that are largely regulated by transcription factors and histone chaperones. Indeed, it was recently shown that a member of the histone chaperone FACT complex is involved in Ig class switching through histone PTM modulation, especially H3K4me3 [26]. The requirement of this particular histone modification in CSR is reminiscent of other programmed recombination events, namely meiotic and VDJ recombinations, in which H3K4me3 is essential for their respective DNA cleavages [27], [28], [29].

Following S region DNA breaks, sealing of the two S region ends is mediated by general DNA repair mechanisms. The major pathway is the error-prone, non-homology-mediated end joining (NHEJ), in which the joining is mediated without long nucleotide microhomology between the paired DNA ends. This type of repair relies heavily on the involvement of the Ku70/80 proteins, which act as anchors for other downstream NHEJ factors to assemble [2], [5]. Another pathway to recombine the cleaved ends is microhomology-mediated joining which depends on the homology of single-stranded overhangs of the two S regions [4].

The DSIF (DRB sensitivity-inducing factor) complex, a transcription factor composed of Spt4 and Spt5, was initially discovered as a factor that rendered RNAPII transcription sensitive to the nucleoside analog 5,6-dichloro-1-β-d-ribofuranosylbenzimidazole (DRB) [30]. DSIF's interaction with the RNAPII complex has been widely reported [31], [32], [33]. DSIF was also shown to be distributed across the body of transcribed genes [34] and to facilitate RNAPII transcription elongation [35], indicating that this complex has a positive effect on transcription. Consistent with its regulatory role in transcription, Spt5 has been reported to regulate histone PTMs that are intimately linked with transcription, such as H2B mono-ubiquitination (ubH2B) and subsequently H3K4me3, through the trans-histone modification pathway [36], [37]. Moreover, DSIF has the potential to inhibit RNAPII progression, notably at the early elongation step through promoter-proximal pausing [31], [38], [39]. However, most of these conclusions are based solely on studies of Spt5, the larger DSIF subunit.

While it was recently reported that Spt5 guides AID to its target sites and is required for CSR [40], the importance of Spt4, the smaller DSIF subunit, in CSR has never been addressed. Our independent screening for transcription elongation factors required for both efficient CSR and histone PTM modulations led us to identify both Spt4 and Spt5 as critical factors for CSR. Using the CH12F3-2A B cell line, which robustly switches to IgA when CIT (CD40L, IL4, and TGFb) is added [41], we showed that depletion of either DSIF subunit abolished CSR. Unexpectedly, however, we found that Spt4 and Spt5 function independently in various phases of CSR, including histone PTM regulation, S region DNA breakage and Sμ cryptic transcript suppression. We also found evidence that Spt4 and Spt5 regulate DNA repair, suggesting that these components play diverse roles to regulate CSR though transcription-coupled processes.

## Results

### Depletion of Spt4 or Spt5 blocks CSR in CH12F3-2A cells

**Figure 1:**
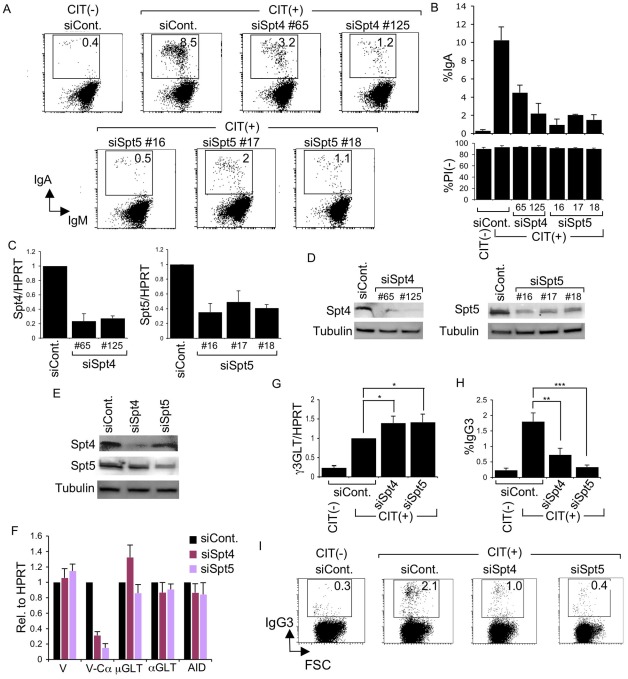
Spt4 and Spt5 are critical for CSR. (A) Flow cytometry (FACS) profile of the percent IgA-switching population, indicated by the number in the box, after introducing the indicated RNAi oligonucleotide under CIT(−) or (+) conditions. (B) Top: Summary of the percent IgA-switching population data derived from the indicated gene knockdown samples. Bottom: Percent of dead cells as determined by PI staining. SD values were determined from three independent experiments. Knockdown efficiency for each gene indicated was analyzed by (C) RT-qPCR and (D) immunoblotting. (E) Immunoblotting of Spt4 and Spt5 derived from the indicated knockdown samples. (F) Various transcripts quantified by RT-qPCR and normalized to HPRT, after introduction of the indicated RNAi oligonucleotide, under the CIT(+) condition. SD values were determined from three independent experiments. (G) After introducing Spt4 or Spt5 RNAi oligonucleotides, γ3GLT was quantified by RT-qPCR normalized to HPRT. SD values were determined from three independent experiments. (H) Summary of IgG3-switching population data derived from the indicated gene-knockdown samples. SD values were determined from three independent experiments. The p-values were calculated using the unpaired two-tailed. Student's *t* test (*, P<0.03; **, P<0.007; ***, P<0.001). (I) FACS profile of the IgG3-switching population after introducing the indicated RNAi oligonucleotide under CIT(−) or (+) conditions.

First, to examine whether both components of the DSIF complex are involved in CSR, we introduced RNAi oligonucleotides into CH12F3-2A cells to knockdown either Spt4 or Spt5. By using multiple siRNA oligonuclotides recognizing different sequences of the target transcripts, flow cytometry analysis showed that depletion of either Spt4 or Spt5 dramatically reduced IgA switching (Figure 1A and 1B, top) without significant cell death (Figure 1B, bottom). RT-qPCR analysis and immunoblotting confirmed that both factors were significantly reduced following the introduction of the specific RNAi oligonucleotides (Figure 1C and 1D).

As all of the RNAi oligonucleotides specific for Spt4 and Spt5 affected CSR dramatically, we selected oligonucleotide #125 and #16 (recognizing Spt4 and Spt5 transcript, respectively) for the subsequent analyses. Spt5 and Spt4 both remained robust even in the absence of their respective counterparts Spt4 and Spt5, although slight reductions in their expressions were visible (Figure 1E).

The absence of either Spt4 or Spt5 did not negatively affect the levels of other transcripts critical for CSR, such as μ-GLT, α-GLT, or AID; the total V_H_ transcripts were also unperturbed (Figure 1F). On the other hand, the V-Cα transcripts, the final CSR products in CH12F3-2A cells, were reduced drastically, confirming that CSR is indeed blocked if either of the two DSIF subunits, Spt4 or Spt5, is depleted (Figure 1F).

Coincidentally, microarray analysis showed augmented IGHG3 (Cγ3) transcript in CH12F3-2A cells in the absence of Spt4 or Spt5 (see below). Using a primer pair recognizing Iγ3 and Cγ3, we confirmed that γ3GLT was indeed enhanced by depleting either of the two DSIF components (Figure 1G). We therefore examined the class-switching to IgG3 upon CIT stimulation in CH12F3-2A cells. Unexpectedly, we observed significant switching to IgG3 within 24 hours of adding CIT, even though the overall IgG3-positive population was much smaller than the IgA-positive population generated within the same time period (Figure 1H and 1I). Nonetheless, the absence of either of the two DSIF components, while augmenting γ3GLT, significantly reduced the IgG3-positive population. We therefore concluded that both components of the DSIF complex are required for efficient CSR to IgG3 as well as IgA in CH12F3-2A cells.

### Spt4 and Spt5 regulate the transcription of distinct genome-wide loci

**Figure 2:**
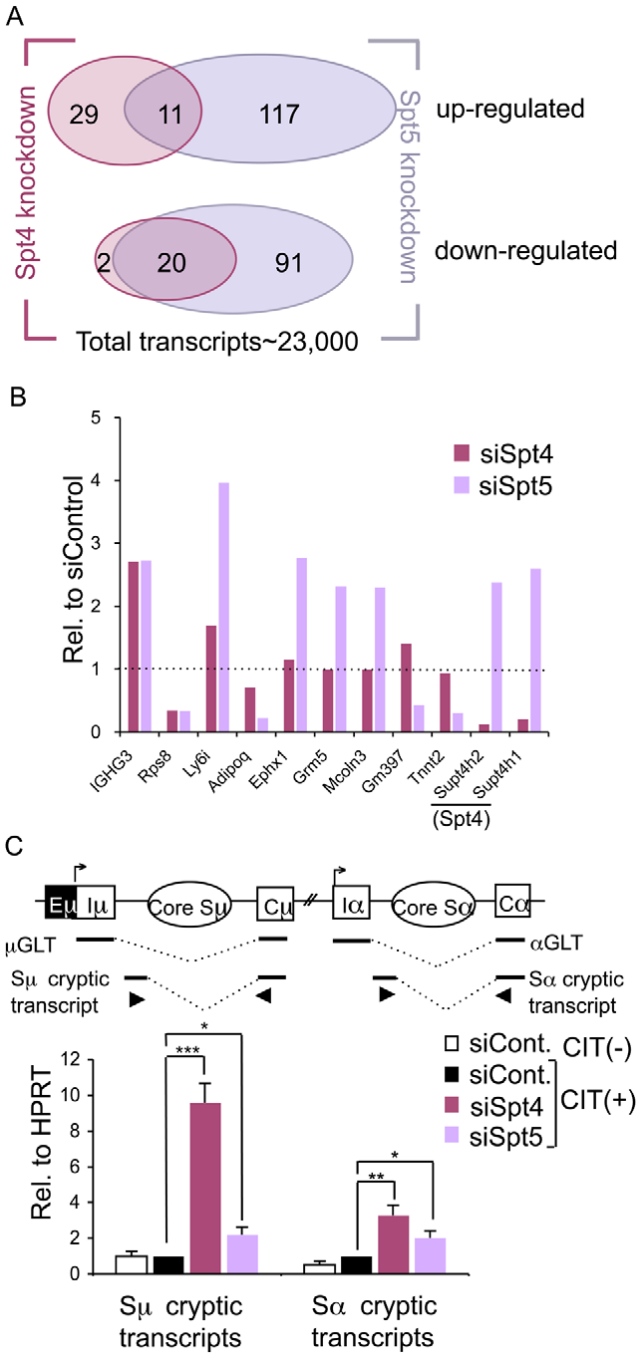
Spt4 and Spt5 regulate small yet distinct sets of transcripts. (A) A Venn diagram showing the number of up- or down-regulated ranscripts by at least 2-fold in the absence of either Spt4 or Spt5, in CIT(+) treated CH12F3-2A cells. (B) Differential expressions of selected genes identified by microarray by either Spt4 or Spt5 knockdown stimulated for 24 hours with CIT; results are presented relative to control, which was set as 1. A complete list is given in Table S1. (C) Top: A schematic diagram of the positions of primers (triangles) used to quantify the cryptic S region transcripts. Bottom: after introducing the RNAi oligonucleotides indicated, the cryptic Sμ and Sα transcripts were quantified by RT-qPCR normalized to HPRT. SD values were determined from three independent experiments. The p-values were calculated using the unpaired two-tailed Student's *t* test (*, P<0.03; **, P<0.004; ***, P<0.0002).

To ascertain that the CSR suppression in the absence of the DSIF complex is not due to the reduced expression of known critical CSR factors, we performed a microarray analysis of global transcripts. This analysis indicated that only a very small number of genes were transcriptionally affected within 48 hours of introducing either Spt4 or Spt5 RNAi oligonucleotides into CIT-stimulated CH12F3-2A cells (Figure 2A). Moreover, the affected transcripts did not seem to code for any critical proteins presently known to be required for efficient CSR (Table S1). Out of about 23,000 transcripts examined, only 40 and 22 transcripts were up- or down-regulated, respectively, by at least 2-fold in the absence of Spt4 (*P*<0.05) (Figure 2A, left circles and Table S1). On the other hand, Spt5 knockdown suppressed 111 transcripts and increased 128 transcripts (Figure 2A, right circles and Table S1). This is consistent with previous studies in HeLa cells, which showed that Spt5 depletion affects only a very small subset of genes [42].

Strikingly, among this small subset of genes whose expression was affected by the depletion of either of the two DSIF components, only 20 and 11 transcripts were commonly down- or up-regulated, respectively, by at least 2-fold (Figure 2A). Several transcripts that show significant difference in the absence of either Spt4 or Spt5 were further quantified by RT-qPCR to confirm that the array result was valid (Figure 2B and Figure S1). These data indicate that Spt4 and Spt5 independently regulate the transcription of a limited number of genes.

Another difference found between Spt4 and Spt5 in the transcriptional regulation came from analysis of the cryptic transcripts that arise within the intronic S region using primer sets detecting intronic Sμ-Cμ and Sα-Cα sequences (Figure 2C, top). These transcripts are generally present at a very low level as compared to GLTs and initiated mainly from cryptic initiator elements (Stanlie, A. unpublished data). Interestingly, Spt4 knockdown dramatically increased the cryptic Sμ transcripts, whereas the effect of Spt5 knockdown is much less (Figure 2C, bottom). However, Spt4 or Spt5 depletion only slightly augmented transcripts initiated from the Sα intronic region; this indicates that there are distinct properties between the donor and acceptor loci. Sμ cryptic transcript suppression is therefore critically dependent on Spt4 but much less on Spt5, further confirming the differential roles of Spt4 and Spt5 in transcriptional regulation.

### Spt4 and Spt5 oppositely regulate S region DNA cleavage and H3K4me3 modification

**Figure 3:**
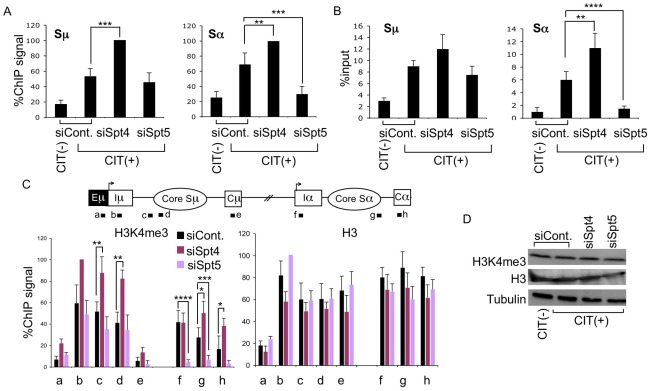
Spt4 and Spt5 differentially control S region DNA cleavage and H3K4me3 status. (A) DNA break assay by γH2AX ChIP using anti-γH2AX antibody were performed in Spt4- or Spt5-knockdown or control samples. Pulled-down DNA was subjected to Sμ- and Sα-specific detection by RT-PCR, normalized to the input DNA signals. The maximum value in each data set was set as 100%. SD values were derived from three independent experiments. (B) DNA break assay performed using biotin-dUTP end labeling method derived from Spt4- or Spt5-knockdown and control samples. Pulled-down DNA was subjected to Sμ- and Sα-specific detection by RT-PCR, normalized to the input DNA. SD values were derived from three independent experiments. (C) Top: schematic diagram of the position of the ChIP assay PCR products. Bottom: the knockdown and control samples indicated were assayed by ChIP, using anti-H3K4me3 and anti-H3 antibodies. Background values from controls with no antibody were subtracted. Values were normalized to the input DNA signals. The maximum value in each data set was set as 100%. SD values were derived from three independent experiments. The p-values were calculated using the unpaired two-tailed Student's *t* test (*, P = 0.05; **, P<0.03; ***, P<0.02; ****, P<0.005). (D) Immunoblotting of histone H3K4me3, H3, and tubulin derived from the indicated knockdown samples.

To analyze the involvement of Spt4 or Spt5 in AID-induced S region DNA cleavage, we conducted two independent assays – ChIP assay of γH2AX focus formation, and *in situ* DNA-end labeling with biotinylated-dUTP to directly measure cleaved DNA ends. Spt4 knockdown did not reduce, but modestly enhanced, γH2AX level and biotinylated-dUTP-labeled DNA fragments in both the Sμ and Sα regions (Figure 3A, 3B). On the other hand, depleting Spt5 significantly reduced both γH2AX foci and biotinylated-dUTP-labeled DNA fragments in the Sα, but not Sμ region (Figure 3A, 3B). These results indicate that Spt4 is dispensable for the DNA cleavage step in both the Sμ and Sα regions, whereas Spt5 is especially critical for introducing DNA breaks in the acceptor Sα region.

Our previous studies of the FACT complex revealed that S region chromatin modifications, particularly H3K4me3, play a critical role in generating AID-induced DNA breaks [26]. Spt5 has been reported to regulate H3K4me3 though the trans-histone modification pathway [36]. To further delineate the importance of Spt4 and Spt5 in H3K4me3 maintenance, we performed ChIP assays using anti-H3K4me3 antibody in the absence of either Spt4 or Spt5. Spt4 knockdown slightly augmented the H3K4me3 modification in both the Sμ and Sα regions as compared to the control, whereas Spt5 depletion drastically reduced the H3K4me3 formation specifically in the Sα but not Sμ region (Figure 3C). H3K4me3 modification levels thus correlated well with DNA breakage efficiency. On the other hand, neither Spt4 nor Spt5 depletion altered the core histone H3 in the Sμ and Sα regions (Figure 3C), suggesting that H3K4me3 depletion in the absence of Spt5 is not ascribed to histone H3 loss, but rather attributed to the inefficiency of the histone modification itself.

We also analyzed the total H3K4me3 and H3 by immunoblotting and found that depletion of Spt4 or Spt5 did not significantly change the cellular levels of these histones (Figure 3D), indicating that the H3K4me3 loss in the absence of Spt5 was not due to a global event, but rather limited to specific loci. These results are consistent with our previous conclusion that the presence of H3K4me3 is a critical determinant for the introduction of S region DNA cleavage in CSR [26]. Furthermore, the present results indicate that distinct transcription factors are required by different S regions to regulate chromatin status and the eventual DNA break. It is striking that Spt4 or Spt5 depletion has opposite effects on both DNA cleavage and H3K4me3 formation at the Sα region.

### The absence of Spt4 or Spt5 inhibits Ku80 accumulation in the Sα region

**Figure 4:**
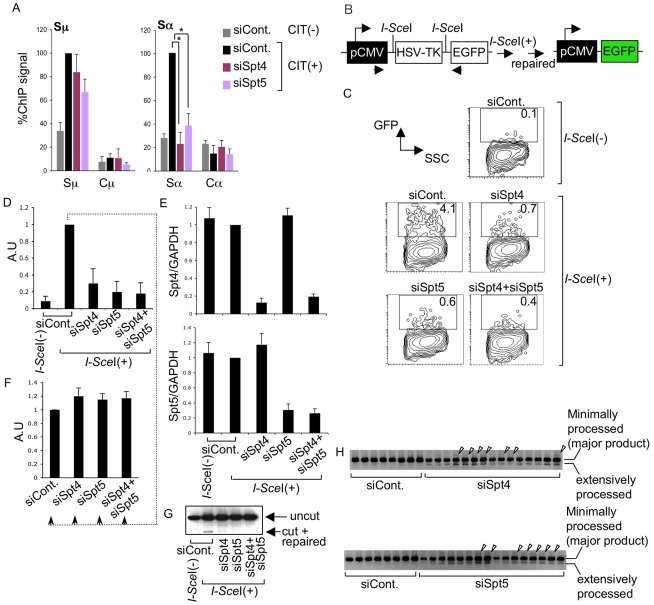
The DSIF complex is required for efficient NHEJ. (A) ChIP assays of the various knockdown and control samples indicated, using anti-Ku80 antibody. Background values from controls with no antibody were subtracted. Values were normalized to the input DNA signals. The maximum value in each data set was set as 100%. SD values were derived from three independent experiments. The p-values were calculated using the unpaired two-tailed Student's *t* test (*, P<0.01). (B) Schematic diagram of the *I-Sce*I-induced NHEJ repair substrate. (C) Percentage of EGFP-positive cells, assessed by FACS analysis 48 hours after co-transfection of *I-Sce*I expression plasmids and the indicated RNAi oligonucleotides into H1299dA3-1 cells. (D) Relative EGFP-positive cells with respect to to *I-Sce*I-treated control cells. SD values were derived from three independent experiments. A.U: arbitrary unit. (E) Knockdown efficiency of the indicated genes was quantified by RT-qPCR. (F) *I-Sce*I(+)-treated control cells from (D) were divided and transfected with the indicated RNAi oligonucleotides (indicated by dotted line); the percent EGFP-positive population was quantified and compared to control samples. SD values were derived from three independent experiments. A.U: arbitrary unit. (G) PCR of genomic DNA products; uncut and repaired fragments derived from control and knockdown samples are indicated by arrowheads. (H) PCR products of repaired genomic DNA fragments derived from the indicated knockdown samples. Arrowheads represent insertions.

Since the absence of Spt4 inhibited CSR but did not reduce the DNA breakage in either donor or acceptor S regions, we suspected a possible CSR repair-phase defect. To investigate possible defects in S region DNA repair in the absence of Spt4 or Spt5, we performed ChIP assays in CH12F3-2A cells using an antibody against Ku80, a protein implicated in the initial phase of NHEJ DNA repair [5]. CIT stimulation enhanced Ku80 accumulation in both the Sμ and Sα regions, while Ku80 was minimally detectable in the Cμ and Cα region (Figure 4A). Depleting either Spt4 or Spt5 did not significantly affect the Ku80 accumulations in the Sμ region, which confirms our observation that DNA cleavage is still detected in the absence of either of the two components. In contrast, depletion of Spt4 or Spt5 was associated with the reduction of Ku80 accumulation in the Sα region. While this result is consistent with the reduction of Sα DNA cleavage by Spt5 knockdown, it was somewhat unexpected because Spt4 knockdown still gave rise to robust DNA breakage. This finding led us to suspect that Spt4 may be involved in the DNA repair phase of CSR.

### The DSIF subunits are required in the DNA repair pathway

To confirm that Spt4 is really required for DNA repair but not cleavage in CSR, we used an artificial NHEJ substrate construct to directly assay Spt4 and Spt5 function in DNA repair as CSR utilizes the general DNA repair pathway [5]. The construct can be cleaved by *I-Sce*I endonuclease at two *I-Sce*I sites flanking a TK cassette, which is actively transcribed under the control of the pCMV promoter (Figure 4B). The joining of two DNA ends with minimal homology sequences brings a GFP cassette close to the promoter, thus initiating its expression. Repair efficiency can be measured by monitoring the GFP-positive cell population by FACS [43].

The introduction of *I-Sce*I expression plasmids into H1299dA3-1 human lung cancer cells carrying this NHEJ construct induced GFP expression (Figure 4C and 4D). The absence of Spt4, Spt5 or both drastically reduced the GFP-positive cell population as compared to the control level (Figure 4C and 4D). RT-qPCR analysis confirmed that the Spt4 and Spt5 gene knockdown was efficient (Figure 4E).

To ascertain that the reduction in GFP-positive cells was not due to transcription inhibition in the pCMV reporter construct, GFP-positive cells expressing *I-Sce*I (indicating they had accomplished NHEJ) were subjected to Spt4, Spt5 or double knockdown. As shown in Figure 4F, the GFP expression was unaltered in the absence of Spt4 and/or Spt5, suggesting that Spt4 and Spt5 do not regulate the GFP reporter's transcription efficiency. Moreover, direct PCR measurement of the ligated DNA ends revealed that the junction signal intensity was reduced in samples treated with Spt4 and/or Spt5 RNAi oligonucleotides (Figure 4G). However, in addition to the major band expected, bands with slightly higher or lower molecular weights were also present, specifically in samples depleted of Spt4 or Spt5 (Figure 4H). These bands were excised and sequenced to identify their origins. While the majority of the PCR products corresponded to the appropriately repaired band, we also obtained clones with long insertions and extensive resections in the products derived from Spt4 or Spt5 knockdown samples (Figure S2), suggesting that the NHEJ repair was compromised. These results revealed that both Spt4 and Spt5 are involved in NHEJ repair of the transcribed locus.

**Figure 5:**
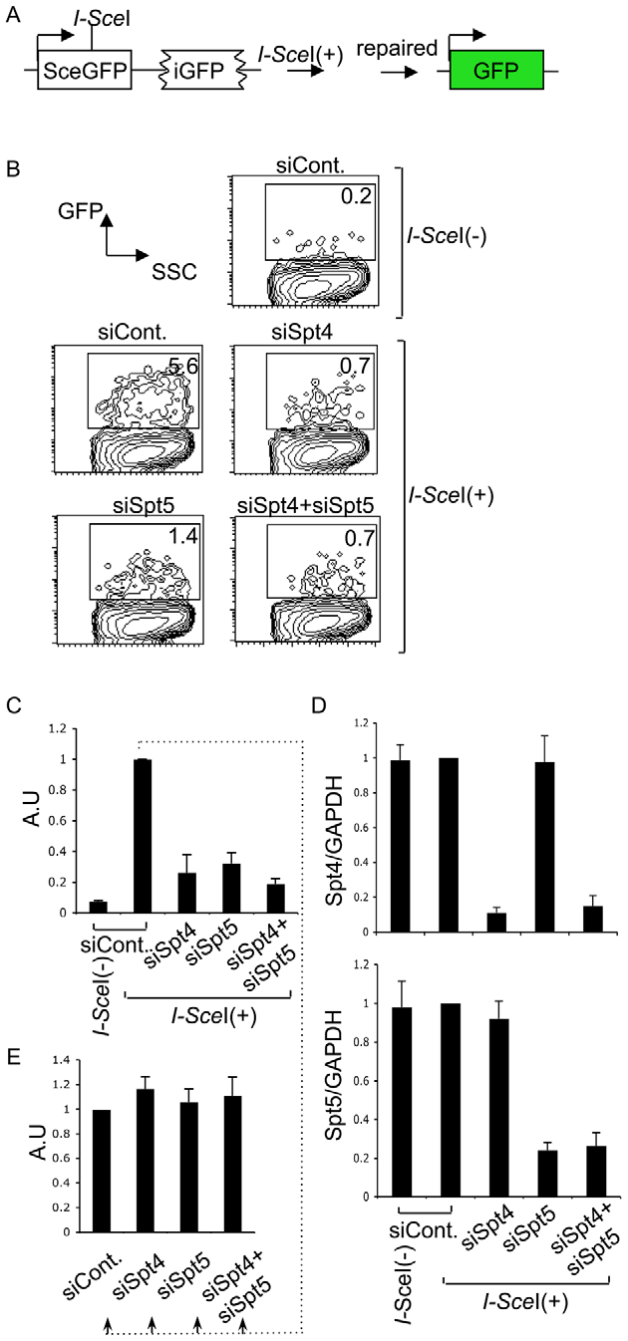
The DSIF complex is also required for efficient homologous recombination. (A) Schematic diagram of the *I-Sce*I-induced homologous recombination substrate. (B) Percent of GFP-positive cells assessed by FACS analysis 48 hours after co-transfection of *I-Sce*I-expressing plasmids and the indicated RNAi oligonucleotides into GM7166VA cells. (C) Relative EGFP-positive cells with respect to to *I-Sce*I-treated control cells. SD values were derived from three independent experiments. A.U: arbitrary unit. (D) Knockdown efficiency of the indicated gene was quantified by RT-qPCR. (E) *I-Sce*I(+)-treated control cells from (C) were divided and transfected with the indicated RNAi oligonucleotides (indicated by dotted line); the percentage of GFP-positive cells was quantified and compared to control samples. SD values were derived from three independent experiments. A.U: arbitrary unit.

We next examined whether Spt4 or Spt5 plays a role in the repair by homologous recombination, again using a synthetic recombination substrate (Figure 5A). This construct is composed of two tandem GFP genes with different mutations: the upstream SceGFP contains the *I-Sce*I recognition site and two in-frame stop codons, and the downstream internal GFP (iGFP) has 5′ and 3′ deletions. *I-Sce*I expression introduces DSBs in SceGFP and triggers repair by gene conversion, using the downstream iGFP as a template [44], [45]. Successful repair can be monitored by GFP expression. The introduction of *I-Sce*I-expression plasmids into GM7166VA cells (derived from a NBS patient) carrying the recombination assay GFP construct robustly induced GFP, which we assayed by FACS (Figure 5B and 5C). However, the GFP-positive population was significantly reduced in the absence of Spt4, Spt5 or both (Figure 5B). RT-qPCR analysis confirmed the gene knockdown efficiency (Figure 5D). We also confirmed that the absence of Spt4 and/or Spt5 did not negatively regulate the transcriptional status of the repaired GFP reporter substrate, using a method similar to the above-mentioned NHEJ assay (Figure 5E). The identical repair assay system conducted in CH12F3-2A cell line gave a similar effect by Spt4, Spt5 or double knockdown (Figure S3A and S3B). Moreover, in the absence of either Spt4 or Spt5, CH12F3-2A cells were more sensitive to ionizing radiation (Figure S3C), whose repair grossly depends on NHEJ [46]. The result is consistent with studies conducted in yeast [47]. Taken together, our findings indicate that Spt4 and Spt5 have essential functions in the DNA repair phase of CSR.

## Discussion

### Spt5, but not Spt4, modulates H3K4me3 formation and DNA breaks in the Sα region

In the present study, we showed that while both components of the DSIF complex, Spt4 and Spt5, are essential for Ig class switching, they have distinct roles in transcriptional regulation, histone PTM maintenance and S region DNA cleavage. Spt5 has been reported to regulate H3K4me3 through the trans-histone modification pathway initiated by H2B ubiquitination (ubH2B) [36], [37]. Interestingly, depletion of a ubiquitin ligase Bre1, which is required for ubH2B leading to efficient H3K4me3 formation, affects the H3K4me3 level specifically in the Sα region but not the Sμ region [26]. Consistent with this finding, we showed that Spt5 depletion strongly affected H3K4me3 and DNA breakage in the Sα region but had relatively little effects in the Sμ region. Spt4 knockdown, on the other hand, slightly augmented DNA breakage in both the Sμ and Sα regions, and slightly increased the H3K4me3 levels. Taken together, these findings indicate that Spt4 and Spt5 differentially regulate histone PTMs, most likely those associated with the ubH2B trans-histone modification cascade. Interestingly, we found that Spt5, but not Spt4 knockdown slightly reduced the AID protein amount without affecting its transcript (Figure S5). As Spt5 and AID have been shown to interact with each other [40], it is possible that Spt5 might directly regulate the stability of this mutator protein. However, since Sμ DNA break formation is unperturbed in the absence of Spt5, the slight depletion of AID expression *per se* is unlikely to be the cause of CSR blockade.

The results presented in this report further strengthen our previous conclusion that H3K4me3 in the S regions provides the histone mark for DNA cleavage sites in CSR [26]. It is intriguing that both VDJ and meiotic recombination also utilize the same histone modification for their respective DNA cleavages [27], [28], [29]. It should be stressed that H3K4me3 is required, but may not be sufficient for the cleavage target determination. An attractive explanation is that a combinatorial readout between H3K4me3 and other histone PTMs or variants, in conjunction with non-B DNA or other specific DNA structures, might be required to recruit a CSR recombinase (i.e. either Top1 or AID itself). In any case, our data support the idea that the presence of nucleosomes and their associated modifications are critical for DSB formation *in vivo*.

### Spt4 and Spt5 have distinct roles in transcription regulation

Interestingly, even though Spt4 and Spt5 are well-established transcription factors, their depletion in CH12F3-2A cells did not affect known critical transcripts and key players of CSR. Spt4 or Spt5 knockdown affected a relatively small subset of transcripts of mostly non-overlapping loci, causing either up- or down- regulation. Moreover, only Spt4 depletion strongly enhanced the Sμ cryptic transcripts, which are initiated from the intronic region; this is reminiscent of the pervasive transcripts observed in the yeast system [48]. It is therefore likely that Spt4 and Spt5 function separately in most of the actively transcribed loci, although they may form the DSIF complex for other functions or loci.

### Distinct features of the donor and recipient S regions

Our data point out distinctive features of the donor Sμ and acceptor Sα switch loci, as summarized in Table S2. Although this distinction has never been clearly defined, previous research has hinted at this possibility. Suv39h1, the methyltransferase responsible for H3K9 trimethylation, specifically promotes switching to IgA but not to other Ig isotypes [49]. Moreover, it was recently reported that a deficiency of the transcription factor Ikaros increases class switching to IgG2b and IgG2a, with a concomitant reduction in all other isotypes; this is achieved by modulating the chromatin status and the transcriptional competency of the γ2b or γ2a genes [50].

These results suggest that different S regions are distinctly regulated. Indeed, using the underlying sequences of Sμ and Sα to predict the nucleosomal occupancy of the loci, we observed a stark contrast in nucleosome distribution in the two regions, especially at the promoter; Iμ promoters have a clearly demarcated nucleosome-free region (NFR), while Iα promoters have no clear NFR boundary (Figure S4). This analysis correlates perfectly with our ChIP data of histone H3 (Figure 3C, compare position ‘a’ and ‘f’). The distinct nucleosomal occupancies, coupled with discrete promoters and chromosomal architecture, might eventually recruit different transcription factors and, in turn, histone PTMs, subsequently dictating the genes' regulatory strategy. It is therefore useful to think of different switch loci as independent entities, regulated distinctly and modulated by various transcription-associated processes.

### Roles of Spt4 and Spt5 in DNA repair are required for CSR

We unexpectedly demonstrated the important roles for Spt4 and Spt5 in DNA repair, using assays with artificial constructs specific to NHEJ and homologous recombination. While Spt4 and Spt5 are not equally necessary for DNA cleavage, they seem to be similarly required for DNA repair; therefore, it is possible that Spt4 and Spt5 may function as a complex for efficient DNA repair. Since Spt4 depletion strongly reduces CSR but not S region cleavage, CSR inhibition in the absence of Spt4 is likely due to the inhibition of the repair phase.

Interestingly, earlier studies found that mutants of Spt4 and Spt5 resulted in methyl methanesulfonate (MMS) sensitivity in yeast, indicating a possible role in DNA repair and recombination [47], [51]. Consistent with this possibility, several repair factors, including BRCA1 and Ku80, have been reported to interact with the DSIF complex in human cells [47], [52]. Moreover, DSB repair proteins involved in NHEJ or homologous recombination such as Ku70/80, DNAPKcs, and RAD51 associate with the RNAPII complex [53]. The interaction between these repair factors and RNAPII transcription elongation machineries suggests that DNA breaks are repaired through transcription-coupled processes at some loci. Such mechanisms have been widely studied in the context of transcription-coupled repair (TCR), in which DNA damage leads to RNAPII arrest, followed by specific factors recruitment to the arrest site, and the lesions are removed by nucleotide excision repair (NER) [54]. Interestingly, deletion of the NER genes ERCC1-XPF is also reported to reduce CSR efficiency [55]. Therefore, the basic idea of TCR might be extended to transcription-coupled homologous recombination and NHEJ, in which efficient repair complexes may interact dynamically with various transcription-associated factors such as Spt4 and Spt5, possibly by modulating the chromatin or histone status. Ultimately, this could increase the stability and residence time of repair factors at the site of DNA damage. It is worth noting that the assay systems employed in our study involve DNA joining in the presence of active transcription, and that efficient CSR repair is likely to be coupled with the transcriptional activity.

CSR's repair phase uses both NHEJ and alternative end-joining systems [2], [3], [4], while homologous recombination is involved in Ig gene conversion. In addition, while AID's exact function in DNA cleavage step is still debated [56], AID is known to be involved in the post-cleavage step of CSR because C-terminally truncated AID mutants can cleave the S regions but cannot complete CSR [12], [13], [14]. If this is the case, it is most likely that AID plays a role in pairing the appropriate ends of the S regions in *cis*. This event requires bending the DNA to bring the two S regions close to each other [57]. It is interesting to note that similar *cis* pairing is required for gene conversion [58]. On the other hand, C-terminally truncated AID can still carry out c-myc-IgH translocation, suggesting that this particular mutation does not inhibit non-specific joining [12]. The present finding that Spt4 and Spt5 are required for NHEJ and homologous recombination suggests that these factors probably remain at the cleaved sites to recruit repair factors until the ends have been successfully joined in CSR.

### Alternative function of Spt5 in determining AID targets

A recent study by Pavri *et al.* showed that Spt5 is required for efficient CSR and associates with more than 9000 AID-targeted loci; these authors assumed that Spt5 is the factor that guides AID to its targets [40]. While our data essentially converge to the conclusion that Spt5 is indeed required for efficient switching, we wondered if this extremely large number of loci bound by Spt5 reflects AID's physiological relevance as a mutator. Such a conundrum is best exemplified by RAG1, which is directly involved in VDJ DNA cleavage and binds preferentially to recombination signal sequences (RSSs) [59]. In addition to these putative RSSs, both mouse and human genomes contain millions of cryptic RSSs that are recognized by RAG proteins [60], [61]. However, current evidence indicates that meaningful RAG-mediated cleavage can occur at some, but most likely not all, of these cryptic sequences [62], [63]. In other words, the propensity for a particular protein to bind to a particular DNA region does not always reflect the true essence of the physiological outcome. More importantly, the target loci of AID and Spt5, while expected to correlate well based on the guiding-factor model do not in fact show a good correlation; by comparing the top 50 targets from both lists only 9 loci are commonly targeted [40], [64]. Our data, on the other hand, suggest that transcription factors like FACT [26], Spt5 (current report) and Spt6 (Begum, N.A; unpublished data) are intimately associated with the S regions, acting primarily as chromatin landscape regulators that in turn promote efficient DNA cleavage. Spt4 and Spt5 are also critical for NHEJ, which is required for CSR.

### Concluding remarks

Collectively, our current data suggest that the DSIF subunits Spt4 and Spt5 can function independently to modulate histone PTM, DNA breakage, and transcription. It remains to be examined whether this dissociation can be observed in contexts other than CSR. It is of note that DSIF's role, especially in RNAPII stalling, was previously studied primarily by focusing on Spt5. Therefore, it remains to be seen whether stalling can be regulated independently at various loci. Finally, since the CSR mechanism is intimately associated with DNA breakage and recombination, often leading to off-target mutations and translocations [65], it is worth investigating the dynamic interactions of various transcription elongation factors that regulate chromatin architecture at non-IgH loci.

## Materials and Methods

### CSR assay and RNAi oligonucleotide transfection

CH12F3–2A cells expressing Bcl2 were cultured and stimulated to induce class switch, as previously described [41]. Cells were subjected to FACS analysis after 24 hours of CIT (CD40L, IL4, and TGFβ) stimulation. FITC-conjugated anti-IgM and PE-conjugated anti-IgA antibodies were used for surface IgM and IgA staining, respectively. To analyze IgG3 switching, cells were prepared by staining with biotinylated anti-IgG3 and allophycocyanin-labeled streptavidin. Dead cells were excluded by propidium iodide staining. All analyses were performed on a FACSCalibur (Becton Dickinson). Electroporation (Amaxa) was used for knockdown experiments and the transfection of various RNAi oligonucleotides (Invitrogen) into CH12F3-2A cells; the cells were cultured for 24 hours, stimulated by CIT, and further cultured for another 24 hours.

### RT–PCR

Total RNA was extracted from cells using TRIzol (Gibco BRL), cDNA was synthesized using Superscript II and Oligo (dT) Primer (Invitrogen), and the real-time PCR reaction was performed using SYBR Green Master Mix (Applied Biosystems) and specific primer pairs.

### Immunoblotting

CH12F3-2A cells were lysed in 1× RIPA lysis buffer containing 10% glycerol and 1% Triton-X-100, and were subjected to immunoblotting following standard protocols.

### Chromatin immunoprecipitation

ChIP assays were performed using ActiveMotif ChIP-IT Express Kit according to the manufacturer's instructions. In brief, 5×10^6^ cells were fixed in the presence of 1% formaldehyde for 5 minutes at room temperature. Glycine was added to a final concentration of 0.125 M to stop the reaction. Cell lysis and sonication yielded a soluble chromatin fraction containing fragmented DNA of 200–500 bp. The lysate was immunoprecipitated by incubation with 2–3 µg of antibody. The pulled-down DNA was detected by real-time PCR normalized to the input. The maximal value in each data set was set as 100%, as described elsewhere [66].

### Biotin–dUTP labeling of DNA–break ends

DNA break assays were performed as described previously [12].

### NHEJ and homologous recombination assay

Lipofectamine 200 reagent (Invitrogen) was used to co-transfect *I-Sce*I-expressing plasmid (pCBASce) and gene-specific RNAi oligonucleotides into either human lung cancer cells (H1299dA3-1) or cells derived from NBS patient repleted with full length human NBS1 (GM7166VA), carrying either NHEJ or homologous recombination artificial repair constructs, respectively (ref. 43, 44). Cells were incubated for 48 hours and analyzed by FACS. For homologous recombination repair analysis in CH12F3-2A cells, the linearized repair construct (linearized by *Xho* I restriction enzyme) was introduced into the cells by electroporation (Amaxa). Stably transfected colonies were picked up after 2 week of selection with puromycin. *I-Sce*I-expressing plasmid was introduced to the cells 24 hour after RNAi introduction. Cells were further incubated for 48 hours and analyzed by FACS.

### Expression array analysis

For the DNA microarray analysis, RNA samples were derived from either Spt4- or Spt5-knockdown CIT-stimulated CH12F3-2A cells. A 3D-Gene Mouse Oligo chip 24k (Toray Industries Inc., Tokyo, Japan) was used (23,522 distinct genes). For efficient hybridization, this microarray has 3 dimensions; that is, it is constructed with a well as the space between the probes and cylinder-stems, with 70-mer oligonucleotide probes on the top. Total RNA was labeled with Cy5 using the Amino Allyl MessageAMP II aRNA Amplification Kit (Applied Biosystems, CA, U.S.A.). The Cy5-labeled aRNA was hybridized for 16 hours using the supplier's protocol (www.3d-gene.com). Hybridization signals were scanned using a ScanArray Express Scanner (PerkinElmer) and were processed by GenePixPro version 5.0 (Molecular Devices). The raw data of each spot was normalized by subtracting the mean intensity of the background signal (determined by all the blank spots' signal intensities with 95% confidence intervals). Raw data intensities greater than 2 standard deviations (SD) of the background signal intensity were considered valid. The signals detected for each gene were subjected to global normalization (the median of the detected signal intensity was adjusted to 30).

### Cytotoxicity assay

For measurement of ionizing radiation sensitivity, CH12F3-2A cells that have been subjected to knockdown for 24-hour period were irradiated with the indicated doses of γ-ray. Cell survival was measured after 2 days by PI staining.

### Accession numbers

The expression array data derived from Spt4 and Spt5 knockdown samples are deposited in GEO under accession number GSE33206.

Information about the antibodies, RNAi oligonucleotides, and primers used are available in Table S3.

## Supporting Information

Figure S1 Spt4 and Spt5 distinctly regulate various transcripts. RT-qPCR analysis of the expression of the indicated mRNAs, derived from either control or Spt4- or Spt5-knockdown samples under CIT-stimulated condition. Results are presented relative to the HPRT mRNA expression. SD values were derived from three independent experiments.
(TIF)

Figure S2 Junction analysis of the NHEJ artificial construct. (A) Analysis of nucleotide sequences at the breakpoint junctions of NHEJ substrate. DNA fragments containing breakpoint junctions were amplified by PCR after transfection of the *I-Sce*I plasmid. PCR products amplified using NHEJfwd. and NHEJrev. primers were subcloned and sequenced. The intact DNA sequence (boxed on the top) is shown. Bold fonts represent DNA duplex, while un-bold fonts represent nucleotide overhang. (B) Various long insertions and extensive resections (underlined) derived from Spt4 or Spt5 knockdown samples. Bold fonts represent *I-Sce*I flanking sequences as shown in (A).
(TIF)

Figure S3 Spt4 and Spt5 are required for efficient DNA repair in CH12F3-2A cells. (A) Percent of GFP-positive cells assessed by FACS analysis 48 hours after transfection of *I-Sce*I-expressing plasmids in the presence of the indicated RNAi oligonucleotides into CH12F3-2A cells containing homologous recombination artificial construct. (B) Relative EGFP-positive cells with respect to *I-Sce*I-treated control cells. SD values were derived from three independent experiments. A.U: arbitrary unit. (C) Sensitivity towards ionizing radiation (γ-ray) of Spt4, Spt5 or control knockdown samples. Cell death curves relative to mock-treated cells as assayed by PI staining is shown. SD values were derived from three independent experiments.
(TIF)

Figure S4 Predicted nucleosomal occupancy of Sμ and Sα loci. The Sμ (accession number AC073553) and Sα (accession number D11468) nucleosomal distributions were predicted and computed based on the nucleosome-DNA sequence interaction model obtained from http://genie.weizmann.ac.il/software/nucleo_prediction.html.
(TIF)

Figure S5 Spt5 knockdown reduces AID protein abundance. Immunoblotting of AID and tubulin derived from either Spt4 or Spt5 knockdown, CIT-stimulated CH12F3-2A cells.
(TIF)

Table S1 Microarray analysis of CH12F3-2A cells after Spt4 or Spt5 knockdown. The fold change in gene expressions in Spt4 or Spt5 RNAi-treated samples relative to control of three replicates was calculated. The genes that were significantly different using a 2-fold cut-off with a P-value of <0.05 are listed.
(XLS)

Table S2 Summary table of the distinct features between Sμ and Sα as well as Spt4 and Spt5 in CH12F3-2A cells. Bre1 is a ubiquitin ligase that mediate mono-ubiquitination of H2B (ubH2B) that in turn regulates H3K4me3 status. NFR: Nucleosome-free Region. The summary data presented here is generated based on samples that have been stimulated by CIT for 24 hour.
(XLS)

Table S3 List of primers, RNAi oligonucleotides and antibodies.
(XLS)
