# Supplementary figures and images for: The DSIF Subunits Spt4 and Spt5 Have Distinct Roles at Various Phases of Immunoglobulin Class Switch Recombination
Source: PLoS Genet. 2012 Apr 26;8(4):e1002675. doi: 10.1371/journal.pgen.1002675 (PMC3343088; doi:10.1371/journal.pgen.1002675)

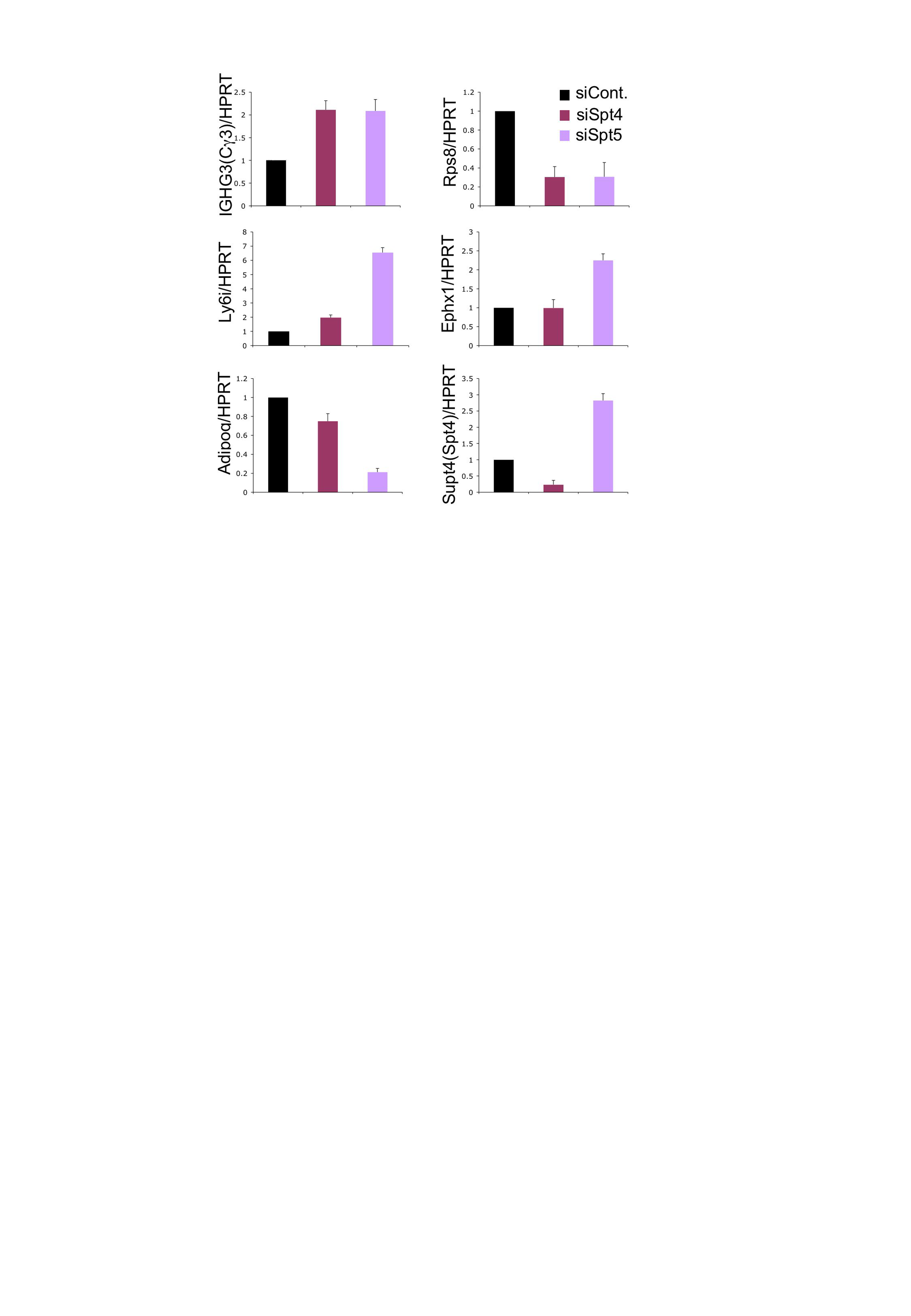

Supplement: Figure S1 — Spt4 and Spt5 distinctly regulate various transcripts. RT-qPCR analysis of the expression of the indicated mRNAs, derived from either control or Spt4- or Spt5-knockdown samples under CIT-stimulated condition. Results are presented relative to the HPRT mRNA expression. SD values were derived from three independent experiments. (TIF) [file pgen.1002675.s001.tif]

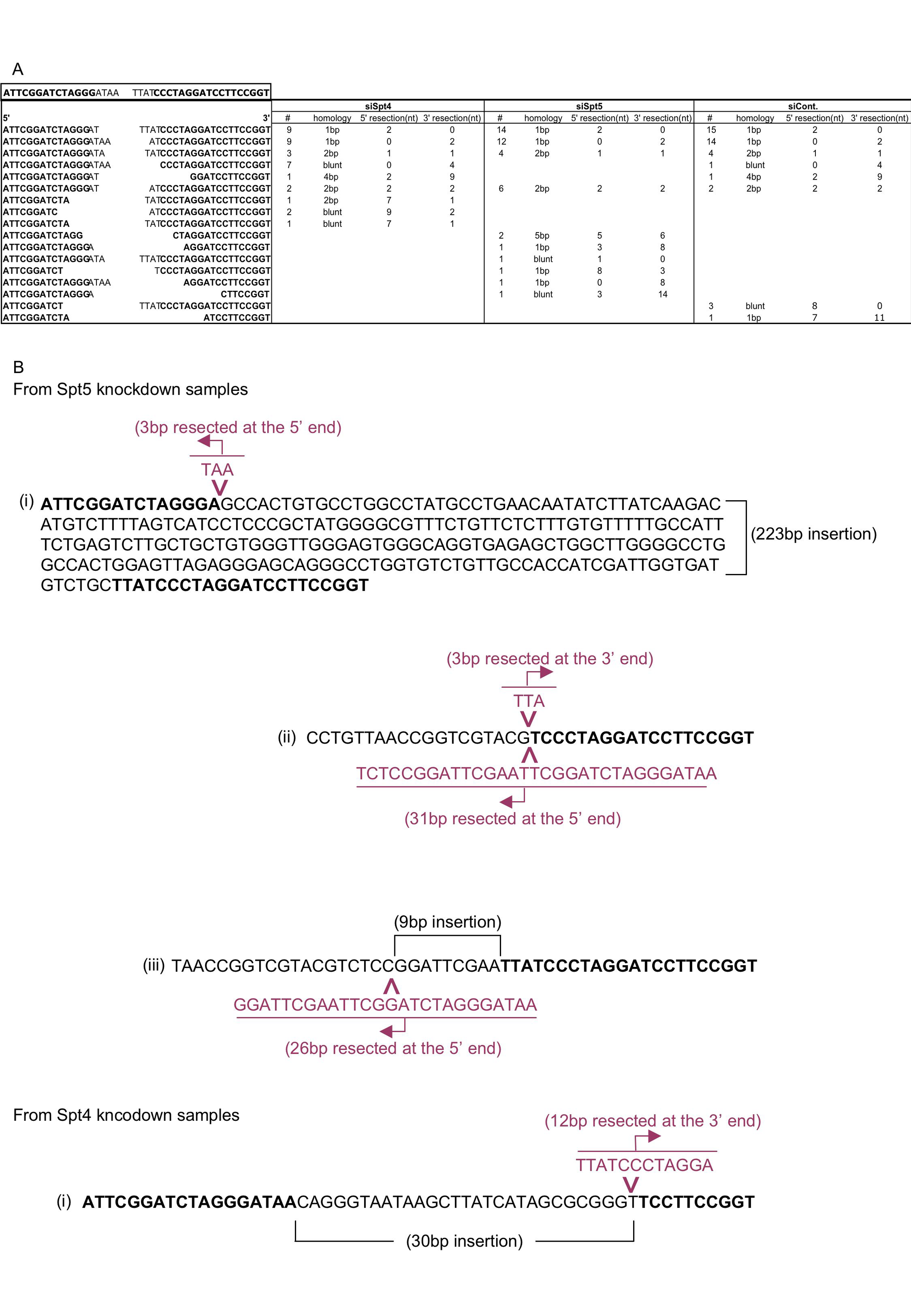

Supplement: Figure S2 — Junction analysis of the NHEJ artificial construct. (A) Analysis of nucleotide sequences at the breakpoint junctions of NHEJ substrate. DNA fragments containing breakpoint junctions were amplified by PCR after transfection of the I-SceI plasmid. PCR products amplified using NHEJfwd. and NHEJrev. primers were subcloned and sequenced. The intact DNA sequence (boxed on the top) is shown. Bold fonts represent DNA duplex, while un-bold fonts represent nucleotide overhang. (B) Various long insertions and extensive resections (underlined) derived from Spt4 or Spt5 knockdown samples. Bold fonts represent I-SceI flanking sequences as shown in (A). (TIF) [file pgen.1002675.s002.tif]

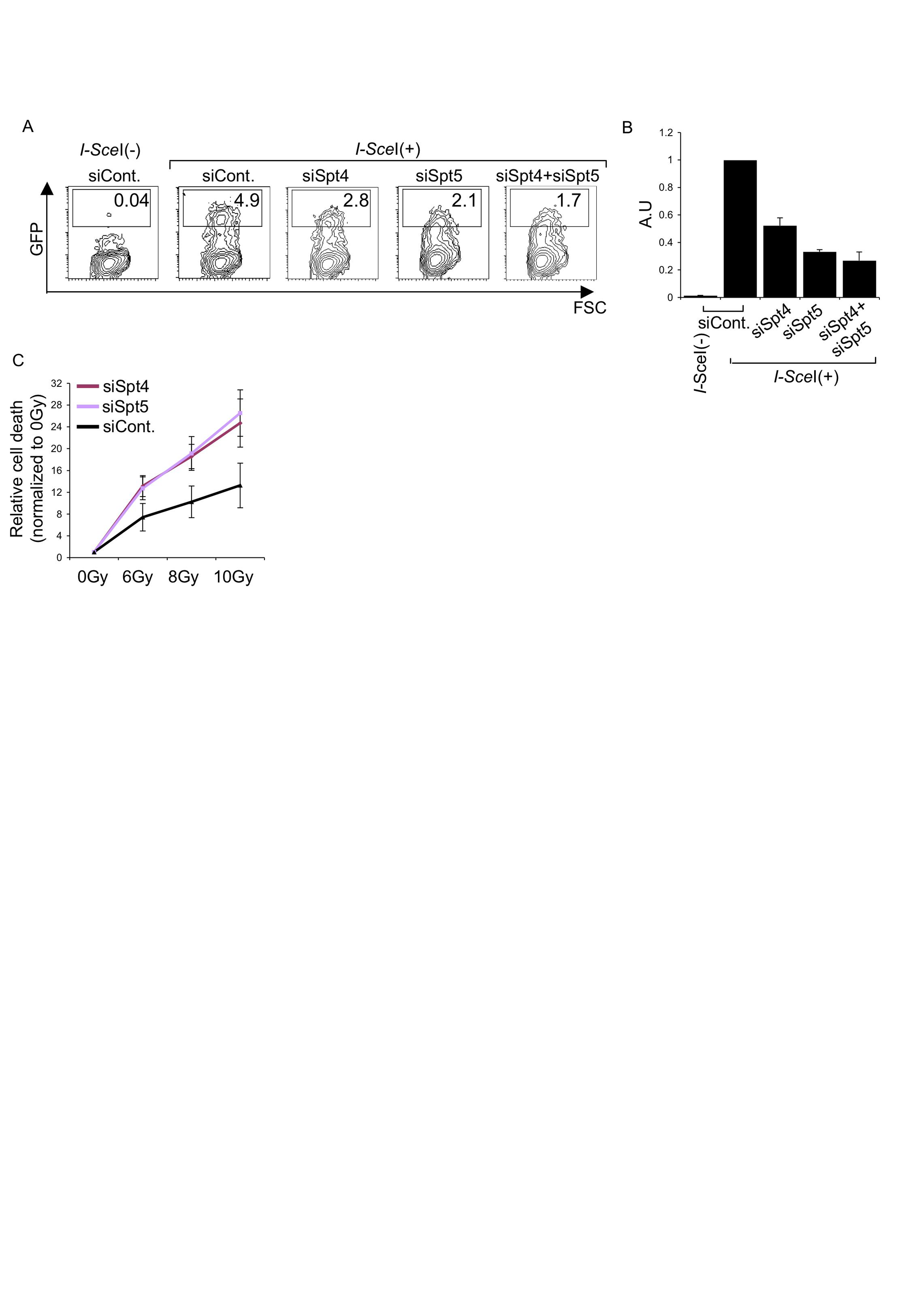

Supplement: Figure S3 — Spt4 and Spt5 are required for efficient DNA repair in CH12F3-2A cells. (A) Percent of GFP-positive cells assessed by FACS analysis 48 hours after transfection of I-SceI-expressing plasmids in the presence of the indicated RNAi oligonucleotides into CH12F3-2A cells containing homologous recombination artificial construct. (B) Relative EGFP-positive cells with respect to I-SceI-treated control cells. SD values were derived from three independent experiments. A.U: arbitrary unit. (C) Sensitivity towards ionizing radiation (γ-ray) of Spt4, Spt5 or control knockdown samples. Cell death curves relative to mock-treated cells as assayed by PI staining is shown. SD values were derived from three independent experiments. (TIF) [file pgen.1002675.s003.tif]

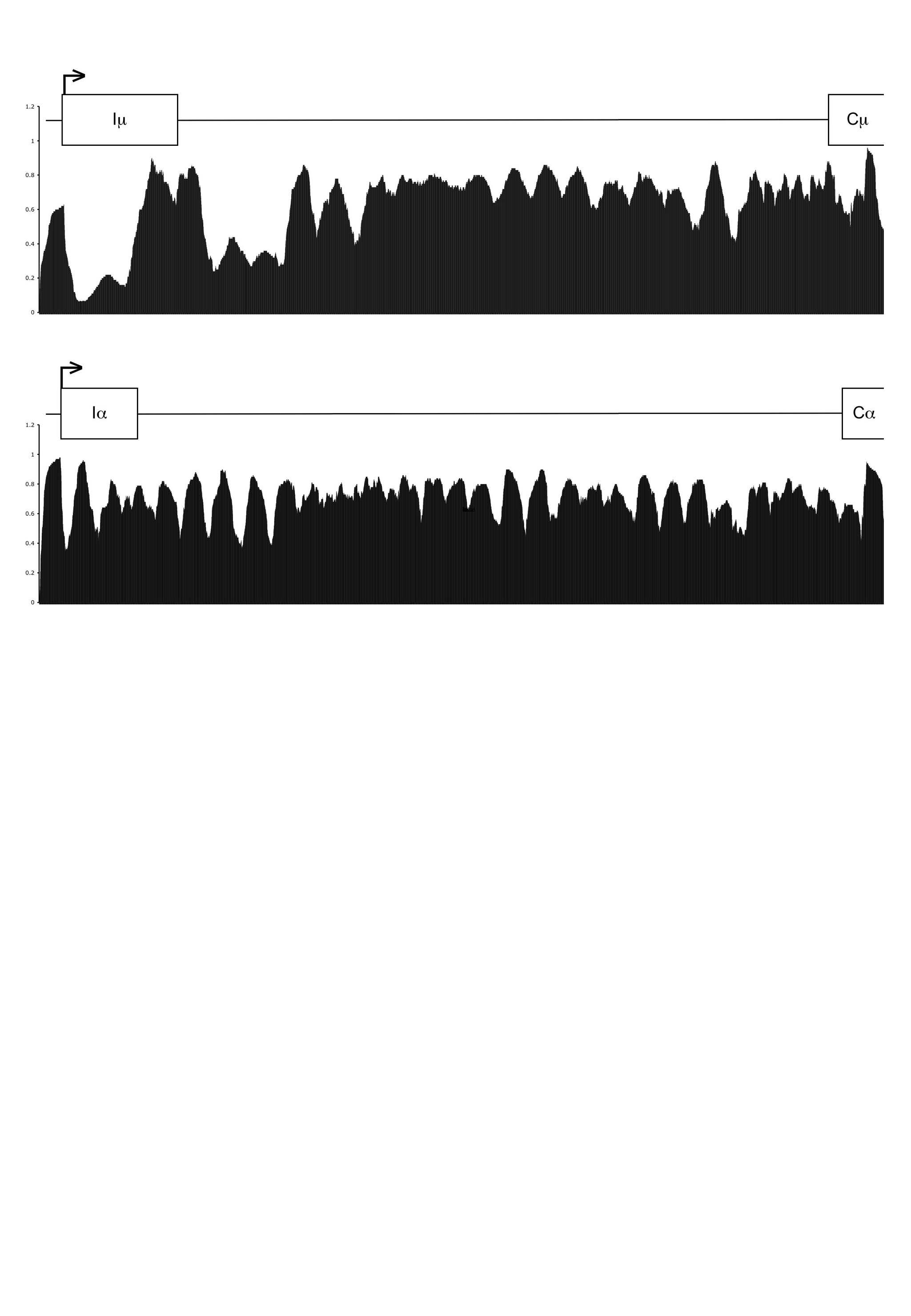

Supplement: Figure S4 — Predicted nucleosomal occupancy of Sμ and Sα loci. The Sμ (accession number AC073553) and Sα (accession number D11468) nucleosomal distributions were predicted and computed based on the nucleosome-DNA sequence interaction model obtained from http://genie.weizmann.ac.il/software/nucleo_prediction.html. (TIF) [file pgen.1002675.s004.tif]

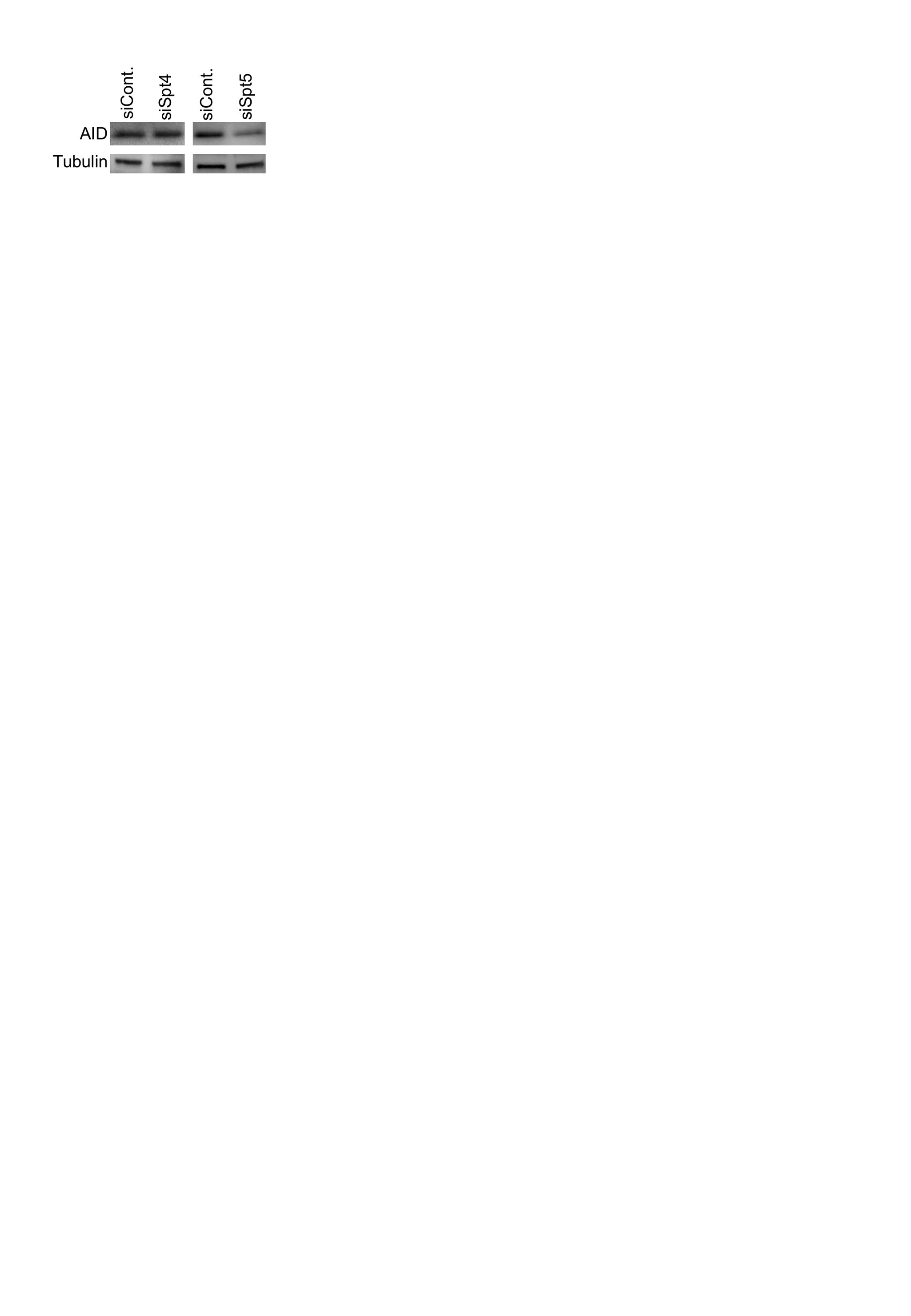

Supplement: Figure S5 — Spt5 knockdown reduces AID protein abundance. Immunoblotting of AID and tubulin derived from either Spt4 or Spt5 knockdown, CIT-stimulated CH12F3-2A cells. (TIF) [file pgen.1002675.s005.tif]
